# Supplementary material for: Varying intensities of chronic stress induce inconsistent responses in weight and plasma metabolites in house sparrows (Passer domesticus)
Source: PeerJ. 2023 Jul 10;11:e15661. doi: 10.7717/peerj.15661 (PMC10340100; doi:10.7717/peerj.15661)
Supplement: Supplemental Information 2 [file peerj-11-15661-s002.docx]

| Bout number | Pre/post | Captivity-only group | Low stress group | Medium stress group | High stress group |
| --- | --- | --- | --- | --- | --- |
| 1 | Pre | 10 | 10 | 10 | 10 |
|  | Post |  | 10 | 10 | 10 |
| 2 | Pre | 10 | 10 | 10 | 10 |
|  | Post |  | 10 | 10 | 10 |
| 3 | Pre | 9 | 9 | 9 | 10 |
|  | Post |  | 9 | 9 | 10 |
| 4 | Pre | 9 | 9 | 8 | 10 |
|  | Post |  | 9 | 8 | 10 |
| 5 | Pre | 9 | 9 | 8 | 10 |
|  | Post |  | 9 | 7 | 9 |
| 6 | Pre | 7 | 9 | 5 | 8 |
|  | Post |  | 9 | 4 | 8 |
| Recovery |  | 6 | 8 | 3 | 7 |
